# Orthogeriatric co-managements lower early mortality in long-lived elderly hip fracture: a post-hoc analysis of a prospective study

**DOI:** 10.1186/s12877-023-04289-z

**Published:** 2023-09-18

**Authors:** Feng Gao, Gang Liu, Yufeng Ge, Zhelun Tan, Yimin Chen, Weidong Peng, Jing Zhang, Xinyi Zhang, Jiusheng He, Liangyuan Wen, Xianhai Wang, Zongxin Shi, Sanbao Hu, Fengpo Sun, Zishun Gong, Mingyao Sun, Maoyi Tian, Shiwen Zhu, Minghui Yang, Xinbao Wu

**Affiliations:** 1 Department of Orthopaedics and Traumatology, Beijing Jishuitan Hospital, Capital Medical University, Beijing, China; 2 Peking University Fourth School of Clinical Medicine, Beijing, China; 3 National Center of Orthopaedics, Beijing, China; 4 School of Population Health, University of New South Wales, Sydney, NSW Australia; 5 The George Institute for Global Health at Peking University Health Science Centre, Beijing, China; 6 Department of Orthopaedics, Beijing Shunyi District Hospital, Beijing, China; 7 Department of Orthopaedics, Beijing Hospital, National Center of Gerontology, Institute of Geriatric Medicine, Chinese Academy of Medical Sciences, Beijing, China; 8 Department of Orthopaedics, Beijing Changping District Hospital, Beijing, China; 9 Department of Orthopaedics, Beijing Liangxiang Hospital, Beijing, China; 10 Department of Orthopaedics, Beijing Anzhen Hospital, Capital Medical University, Beijing, China; 11 School of Public Health, Harbin Medical University, Harbin, China

**Keywords:** Elderly, Long-lived, Hip fractures, Co-management care, Orthogeriatric, Mortality

## Abstract

**Objective:**

To evaluate the clinical effectiveness of orthogeriatric co-management care in long-lived elderly hip fracture patients (age ≥ 90).

**Methods:**

Secondary analysis was conducted in long-lived hip fracture patients between 2018 to 2019 in 6 hospitals in Beijing, China. Patients were divided into the orthogeriatric co-management group (CM group) and traditional consultation mode group (TC group) depending on the management mode. With 30-day mortality as the primary outcome, multivariate regression analyses were performed after adjusting for potential covariates. 30-day mobility and quality of life were compared between groups.

**Results:**

A total of 233 patients were included, 223 of whom completed follow-up (125 in CM group, 98 in TC group). The average age was 92.4 ± 2.5 years old (range 90–102). The 30-day mortality in CM group was significantly lower than that in TC group after adjustments for (2.4% vs. 10.2%; OR = 0.231; 95% CI 0.059 ~ 0.896; *P* = 0.034). The proportion of patients undergoing surgery and surgery performed within 48 h also favored the CM group (97.6% vs. 85.7%, *P* = 0.002; 74.4% vs. 24.5%, *P* < 0.001; respectively). In addition, much more patients in CM group could walk with or without aids in postoperative 30 days than in the TC group (87.7% vs. 60.2%, *P* < 0.05), although differences were not found after 1-year follow-up. And there was no significant difference in total cost between the two groups (*P* > 0.05).

**Conclusions:**

For long-lived elderly hip fracture patients, orthogeriatric co-management care lowered early mortality, improved early mobility and compared with the traditional consultation mode.

## Introduction

Hip fracture in the elderly is a serious fragile fracture, and often associated with low energy falls and decreased bone mineral density [1, 2], which means for osteoporosis patients, even the impact force applied to the hip from a slight lateral fall may result in a hip fracture. Hip fracture brings a huge burden to patients and the national medical system with a high mortality risk, severe decrease in health-related quality of life and the high medical cost [3]. Previous studies have shown that advanced age is associated with increased mortality rates and worse functional recovery after a hip fracture [4].

In recent years, the global population aging process has been accelerating and deepening, the number of the elderly over 90 years old (also known as the long-lived elderly) in China was approximately 1.2 million in 2020. The number of long-lived elderly is rapidly increasing and therewith the number of long-lived elderly hip fracture patients [5]. The long-lived elderly patients frequently have poor cardiopulmonary function, severe osteoporosis, and often suffer from complex comorbidities and functional impairment [6–8], which makes them difficult to tolerate anesthesia and makes the operation more difficult [9]. And they are also more likely to develop postoperative pulmonary infection, deep vein thrombosis (DVT) and other complications [10], which leads to a higher mortality risk. A recent study in Japan [11] showed that age is closely related to the mortality after hip fracture, and when the age is more than 90 years old, the mortality risk of hip fracture will increase sharply, and the incidence of postoperative respiratory complications will increase significantly. How to effectively manage hip fracture patients aged 90 and above has become an urgent problem to be solved.

The efficient management of hip fracture is complex and multifaceted, which involves various aspects such as the management of comorbidity and complication, surgical implementation, rehabilitation, and fracture secondary prevention (anti-osteoporosis treatment and falls prevention) [1, 2, 12, 13]. Orthogeriatric co-management care is defined as involvement of an orthopedic physician and geriatrician in daily trauma care [13, 14]. It is reported in the literature and guidelines that orthogeriatric co-management care can significantly improve the prognosis of elderly hip fracture patients [15–17], but there is a lack of research focusing on the long-lived elderly patients. This study aims to explore the impact of orthogeriatric co-management care on the long-lived elderly hip fracture patients.

## Patients and methods

### Study design and settings

This study was a post-hoc analysis of a prospective multicenter quasi-experimental study [18] comparing the effectiveness of a co-management care mode on older hip fracture patients in China, which continuously recruited hip fracture patients aged 65 and above who were admitted to 6 hospitals in Beijing from November 2018 to November 2019. The study was registered at Clinicaltrials.gov (NCT03184896). Among all the hospitals, one hospital had set up an independent orthogeriatric ward, which adopted orthogeriatric co-management care mode for the perioperative management of elderly hip fracture patients, and was set as CM group; the other five hospitals, which adopted the traditional consultation mode, were set as TC group. We selected the long-lived patients (age ≥ 90) from the two groups, and then retrospectively compared the effects of the two modes on the prognosis of patients.

The inclusion criteria for this study were as follows: 1) Patients age ≥ 90 years old; 2) Patients with hip fracture diagnosed by X-ray and/or CT examination (femoral neck fracture, intertrochanteric fracture and subtrochanteric fracture); and 3) the time from injury to admission is less than 21 days. The exclusion criteria were as follows: 1) Patients with neoplastic pathological fracture; 2) Patients with peri-prosthesis fracture.

The original study was conducted in accordance with the Helsinki Declaration, and was registered at Clinicaltrials.gov. Ethics approvals were granted by our institutional ethics committees. All informed participants provided written consent.

### Intervention and control

Orthogeriatric co-management group (CM group): orthogeriatric co-management care mode was adopted. Upon admission, the patient entered an independent orthogeriatric ward, which is co-managed by orthopedic and geriatric doctors. Key points included: early operation (< 48 h), comorbidity evaluation and management, secondary prevention of fracture, pressure sores prevention, physical therapy and early discharge. Geriatricians were responsible for preoperative evaluation, comorbidity management, prevention of postoperative complications and secondary prevention of fractures (bone protection treatment and fall assessment); and the orthopedic surgeons were responsible for the preparation and execution of the surgical operation. In addition, rehabilitation physicians, nutritionists and nurses also participated in the perioperative management of patients. During the research period, all recruited patients in this hospital received co-management care in the orthogeriatric ward.

Traditional consultation mode group (TC group): traditional consultation mode was used. All patients were admitted to the traditional orthopedic ward and managed by orthopedic surgeons. Physicians and geriatrics can be consulted to assist in diagnosis and treatment if necessary.

### Data collection and outcomes

In the original study, trained nurses from orthopedic ward in each hospital were responsible for patients’ screening, enrolment, and data collection at the baseline and follow-ups. The patient demographic information, pre-operative information, peri-operative information, post-operative information, and the follow up information were recorded. Recruited patients were followed up at three time points via telephone (30 days, 120 days, and 1 year post admission). The last patient follow-up was completed on November 30, 2020. All data was established as a database. We select the data of long-lived patients from the database for analysis.

The primary outcome was 30-day mortality. And secondary outcome variables included in-hospital mortality, 1-year mortality, the proportion of patients undergoing surgery, the proportion of surgery performed within 48 h, hospital length of stay (LOS), 1-year reoperation rate, the incidence of clinical adverse events (Delirium, Stroke, Deep venous thrombosis (DVT), Pneumonia, Urinary tract infection, Cardiac complication, Pressure sores), the total cost in thousand yuan, the mobility and the quality of life (QoL) in 30 days and 1 year post admission.

The EuroQol Five Dimensions (EQ-5D) Questionnaire was used to assess patients’ health related quality of life (HRQoL), which systematically describes the HRQoL of patients from five dimensions, including mobility, self-care, usual activities, pain / discomfort, anxiety / depression. Each dimension contains five levels: having no problems, having slight problems, having moderate problems, having severe problems and being unable to do / having extreme problems. The health status of the five dimensions can be converted into EQ-5D index value through the scoring algorithm. In addition, the questionnaire used a standard 20 cm visual analog scale (VAS) to allow respondents to make self-evaluation of their own health status. The VAS score can be used to represent the overall health status of patients, with a minimum of 0 (the worst) and a maximum of 100 (the best).

### Statistical analysis

Means with standard deviation (SD) or median with quartile range or proportion were used to describe patients’ demographic and clinical characteristics at baseline and follow-up. Student’s t-test or Mann–Whitney U test were adopted to test for continuous variables, while Chi-square test was adopted to test for categorical variables. The continuous secondary outcomes (hospital LOS, total cost, EQ-5D index and EQ-VAS) were compared using multivariable linear regression models, regression coefficient (b) and T value (T) were calculated. The primary outcome (30-day mortality) and other binary secondary outcomes (in-hospital mortality, 1-year mortality, mobility, reoperation, clinical adverse events, etc.) were compared between two groups using multivariable logistic regression models, odds ratios (OR) and 95% confidence interval (95% CI) were calculated. Multivariate analysis included clinically meaningful variables (age, whether co-management care, American Society of Anesthesiologist (ASA) grade) and statistically significant variables: gender, living place, pre-fracture mobility, fracture types. Statistical analysis was performed using the IBM SPSS Statistical Package (version 25) (SPSS Inc., Chicago, IL, USA). All statistical significance was established at* P* < 0.05.

## Results

**Fig.1:**
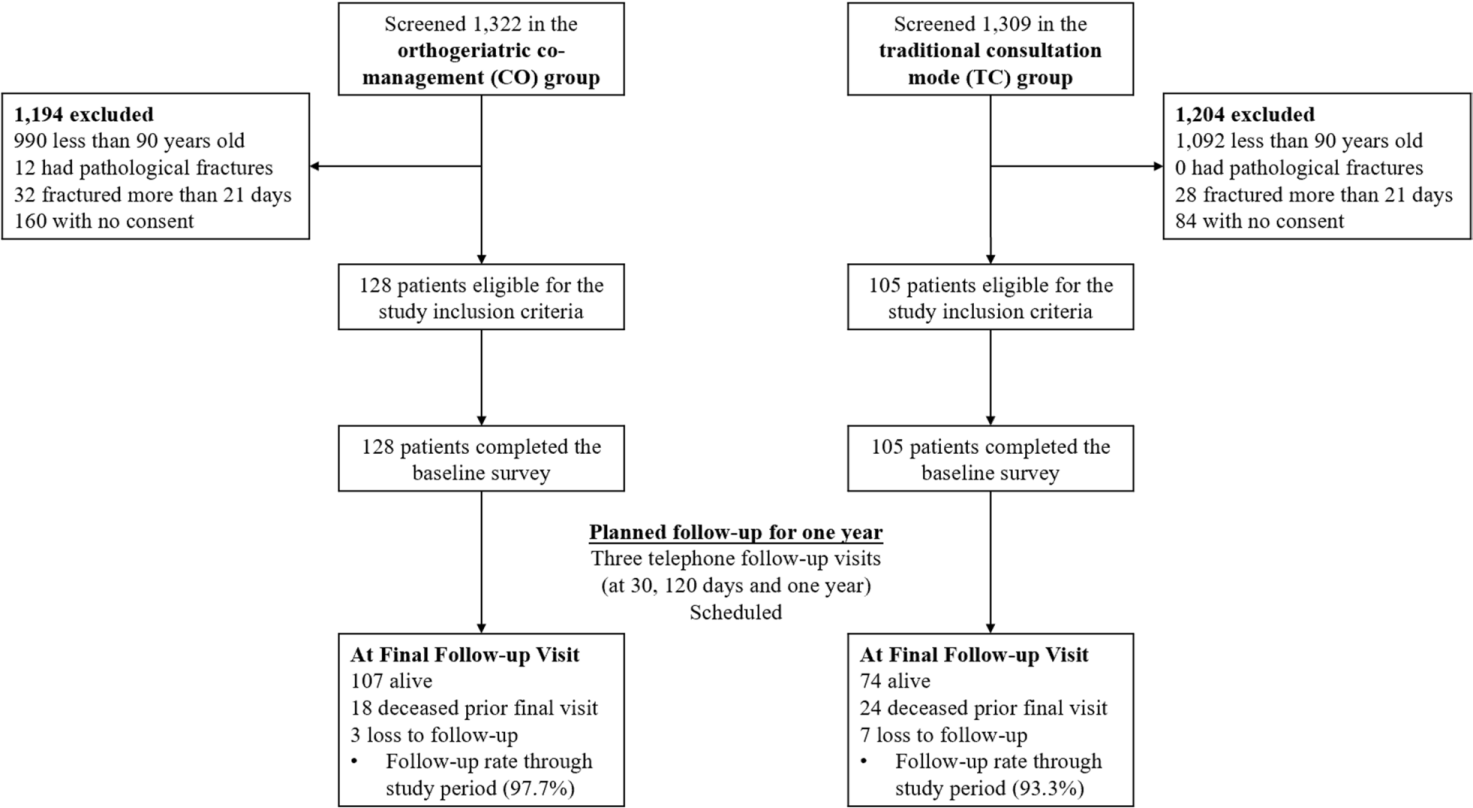
Research flow chart

A total of 233 long-lived elderly hip fracture patients were recruited in this study, with 125 and 98 patients in the CM and TC groups, respectively. 10 cases were lost to follow-up, and the follow-up rate was 95.71%. The research flow chart is shown in Fig. 1. The data of patients with complete follow-up were analyzed.

The average age is 92.42 ± 2.46 years old (range 90–102 years old). There was statistically significant difference in gender, living place, pre-fracture mobility and fracture type (*P* < 0.05). The baseline characteristics of recruited patients and the comparisons of TC and CM group are shown in Table 1.

**Table 1:** Comparison of baseline characteristics between two groups

	Total (*n* = 223)	TC group (*n* = 98)	CM group (*n* = 125)	Statistics	*p* value
Age in years, median (IQR)	92 (91, 94)	92 (90, 93.25)	92 (91, 94)	-0.307	0.759
Gender, n (%)				3.958	0.047
Male	73 (32.74)	39 (39.80)	34 (27.20)		
Female	150 (67.26)	59 (60.20)	91 (72.80)		
Smoking history, n (%)	34 (15.25)	14 (14.29)	20 (16.00)	0.125	0.724
Alcohol drinking, n (%)	9 (4.04)	5 (5.10)	4 (3.20)	0.140	0.709
Living place, n (%)				35.864	< 0.001
Rural area	184 (82.51)	64 (65.31)	120 (96.00)		
Urban area	39 (17.49)	34 (34.69)	5 (4.00)		
Pre-fracture mobility, n (%)				31.922	< 0.001
Independent	83 (37.22)	28 (28.57)	55 (44.00)		
Walking aid	89 (39.91)	30 (30.61)	59 (47.20)		
Non-ambulant	51 (22.87)	40 (40.82)	11 (8.80)		
Fracture type, n (%)				4.948	0.026
Femoral neck fracture	77 (34.53)	26 (26.53)	51 (40.80)		
Intertrochanteric/Subtrochanteric fracture	146 (65.47)	72 (73.47)	74 (59.20)		
ASA grade, n (%)				0.252	0.616
Grade 1–2	105 (47.09)	48 (48.98)	57 (45.60)		
Grade 3–4	118 (52.91)	50 (51.02)	68 (54.40)		
Hypertension, n (%)	125 (56.05)	53 (54.08)	72 (57.60)	0.276	0.599
Diabetes mellitus, n (%)	31 (13.90)	15 (15.31)	16 (12.80)	0.288	0.591
Coronary heart disease, n (%)	58 (26.01)	24 (24.49)	34 (27.20)	0.210	0.647
Dementia, n (%)	19 (8.52)	5 (5.10)	14 (11.20)	2.621	0.105

*TC* traditional consultation mode, *CM* orthogeriatric co-management, *ASA* American Society of Anesthesiologists

The 30-day mortality of TC group and CM group were 10.20% (10/98) and 2.40% (3/125) respectively, which in CM group was significantly lower than that in TC group (*P* < 0.05). After adjusting for gender, age, living place, pre-fracture mobility, fracture type, ASA grade, and whether co-management care, there was still significant statistical difference between the two groups (OR = 0.231, 95%CI 0.059 ~ 0.896, *P* = 0.034) (Table 2).

**Table 2:** Comparison of major outcome variables between two groups

	Total (*n* = 223)	TC group (*n* = 98)	CM group (*n* = 125)	Statistics	*p* value	OR / b	95% CI / T	Adjusted *p* value*
30-day mortality, n (%)	13 (5.83)	10 (10.20)	3 (2.40)	6.094	0.014	0.231	(0.059 ~ 0.896)	0.034
In-hospital mortality, n (%)	6 (2.69)	5 (5.10)	1 (0.80)	2.414	0.120	0.071	(0.008 ~ 0.663)	0.020
1-year mortality, n (%)	42 (18.83)	24 (24.49)	18 (14.40)	3.658	0.056	-	-	0.381
Treated with surgery, n (%)	206 (92.38)	84 (85.71)	122 (97.60)	11.020	0.001	7.313	(2.015 ~ 26.546)	0.002
Performed surgery within 48 h from admission, n (%)	117 (52.47)	24 (24.49)	93 (74.40)	54.869	< 0.001	12.980	(6.240 ~ 26.999)	< 0.001
Hospital LOS, mean ± SD (days)	8.97 ± 7.32	11.92 ± 9.15	6.66 ± 4.27	5.255	< 0.001	-6.137	-6.128	< 0.001
Reoperation within 1 year, n (%)	3 (1.35)	1 (1.02)	2 (1.60)	< 0.001	1.000	-	-	0.709
Total clinical adverse events, n (%)	23 (10.31)	12 (12.24)	11 (8.80)	0.705	0.401	-	-	0.401
Delirium, n (%)	2 (0.90)	1 (1.02)	1 (0.80)	-	1.000	-	-	0.922
Stroke, n (%)	1 (0.45)	0 (0)	1 (0.80)	-	1.000	-	-	0.375
DVT, n (%)	5 (2.24)	3 (3.06)	2 (1.60)	0.076	0.783	-	-	0.336
Pneumonia, n (%)	6 (2.69)	2 (2.04)	4 (3.20)	0.013	0.909	-	-	0.368
Urinary tract infection, n (%)	3 (1.35)	3 (3.06)	0 (0)	1.915	0.166	-	-	0.996
Cardiac complication, n (%)	3 (1.35)	0 (0)	3 (2.40)	0.919	0.338	-	-	0.997
Pressure sores, n (%)	6 (2.69)	4 (4.08)	2 (1.60)	0.518	0.472	-	-	0.104
Total cost in thousand yuan, median (IQR)	58.8 (47.4, 70.8)	55.8 (43.0, 72.3)	59.7 (52.9, 66.0)	-1.229	0.219	-	-	0.323

*TC* traditional consultation mode, *CM* orthogeriatric co-management, *LOS* length of stay, *DVT* Deep venous thrombosis, *OR* odds ratio, *95% CI* 95% confidence interval

^*^Multivariate analysis included gender, age, living place, pre-fracture mobility, fracture type, ASA grade, and whether co-management care

There was no significant difference in the in-hospital mortality (CM group 0.80% vs. TC group 5.10%, *P* > 0.05), but after adjustment, the orthogeriatric co-management care has significant protective influence on in-hospital mortality (OR = 0.071, 95%CI 0.008 ~ 0.663, *P* = 0.020). The proportion of patients undergoing surgery (97.60% vs. 85.71%), the proportion of surgery performed within 48 h (74.40% vs. 24.49%), hospital LOS (6.66 ± 4.27d vs. 11.92 ± 9.15d), 30-day mobility (independent: walking aid: non-ambulant 5.74%: 81.97%: 12.30% vs. 3.41%: 56.82%: 39.77%) and 30-day EQ-5D index (0.53 ± 0.24 vs. 0.36 ± 0.28) in CM group were statistically significantly better than those in TC group (*P* < 0.05). After adjustment, the orthogeriatric co-management care also has significant influence on in-hospital mortality (CM group 0.80% vs TC group 5.10%, OR = 0.071, 95%CI 0.008 ~ 0.663, *P* = 0.020), the proportion of patients undergoing surgery (OR = 7.313, 95%CI 2.015 ~ 26.546, *P* = 0.002), the proportion of surgery performed within 48 h (OR = 12.980, 95%CI 6.240 ~ 26.999, *P* < 0.001), hospital LOS (b = -6.137, T = -6.128, *P* < 0.001), 30-day mobility (OR = 0.176, 95%CI 0.073 ~ 0.421, *P* < 0.001) and 30-day EQ-5D index (b = 0.181, T = 4.435, *P* < 0.001). And there was no significant difference in the incidence of clinical adverse events (*P* > 0.05), 1-year mortality (*P* = 0.381), total cost (*P* = 0.323), 1-year mobility (*P* = 0.267) and 1-year QoL (*P* = 0.234) between the two groups. The complete comparison of secondary outcome variables and the results of multivariate analysis can be seen in Tables 2 and 3. There were 3 cases of reoperation, including 1 case of prosthesis dislocation after hip replacement in the TC group and 1 case of artificial femoral head replacement and 1 case of periprosthetic fracture in the CM group.

**Table 3:** Comparison of mobility and QoL between two groups of patients survived 30 days and 1 year after admission

	Total	TC group	CM group	Statistics	*p* valu*e*	OR/b	95%CI/T	Adjusted *p* value*
30-day mobility, n (%)				21.321	< 0.001			
Independent	10 (4.76)	3 (3.41)	7 (5.74)			0.090	(0.013 ~ 0.619)	0.014
Walking aid	150 (71.43)	50 (56.82)	100 (81.97)			0.176	(0.073 ~ 0.421)	< 0.001
Non-ambulant	50 (23.81)	35 (39.77)	15 (12.30)			Ref	Ref	Ref
1-year mobility, n (%)				2.639	0.267			
Independent	39 (21.55)	16 (21.62)	23 (21.50)			0.779	(0.197 ~ 3.081)	0.722
Walking aid	123 (67.96)	47 (63.51)	76 (71.03)			0.709	(0.209 ~ 2.403)	0.581
Non-ambulant	19 (10.50)	11 (14.86)	8 (7.48)			Ref	Ref	Ref
30-day EQ-5D index, mean ± SD	0.45 ± 0.27	0.36 ± 0.28	0.53 ± 0.24	-4.506	< 0.001	0.181	4.435	< 0.001
1-year EQ-5D index, mean ± SD	0.69 ± 0.29	0.63 ± 0.36	0.72 ± 0.24	-1.910	0.059	0.091	1.194	0.234
30-day EQ-VAS, mean ± SD	66.78 ± 16.87	64.82 ± 19.94	68.20 ± 14.18	-1.359	0.176	0.027	0.371	0.711
1-year EQ-VAS, mean ± SD	74.6 ± 17.04	74.15 ± 20.92	73.99 ± 13.86	0.056	0.955	-0.033	-0.423	0.673

*TC* traditional consultation mode, *CM* orthogeriatric co-management, *QoL* quality of life, *EQ-5D* EuroQol Five Dimensions Questionnaire, *VAS* Visual Analog Scale, *OR* odds ratio, *95% CI* 95% confidence interval

^*^Multivariate analysis included gender, age, living place, pre-fracture mobility, fracture type, ASA grade, and whether co-management care

## Discussion

In the present study, the CM group was better than the TC group in terms of 30-day mortality, in-hospital mortality, and 30-day EQ-5D index, with statistically significant differences. Furthermore, the proportion of patients undergoing surgery and the proportion of surgery performed within 48 h in CM group were higher, and the hospital LOS was shorter. There was no significant difference in 1-year mortality, incidence of clinical adverse events, 1-year reoperation rate, and 1-year QoL between the two groups.

Mortality is an important reported outcome variable in literature indicating the clinical efficacy of hip fracture in the elderly [10, 11, 19–25]. Therefore, the 30-day mortality was selected as the primary outcome variable in this study. In our study, the 30-day mortality rate in long-lived elderly patients of the TC group was 10.20%, which was in concordance with the literature (10.9–15.2%) [19, 26, 27], significantly higher than that observed in the CM group (2.40%). It was reported previously that orthogeriatric co-management care reduces the early mortality of elderly hip fracture patients [15–19, 28–30], 6 weeks in some studies [19]. There are also some literature reported that orthogeriatric co-management care can reduce the mortality of long-lived elderly patients whose age is over 90, which is in line with the results of this study [15–17, 30]. Pneumonia and circulatory system diseases were the commonest causes of death in our study, similar to that reported in other study [31, 32]. The lower early mortality amongst patients treated by orthogeriatric co-management care probably resulted from some different factors in some related literature [19]: First, the medical problems in long-lived patients are often of geriatric nature and are therefore better treated by geriatrist. Comorbidities like cerebrovascular or kidney disease and ICU admission were significant risk factors that increased mortality after osteoporotic hip fractures [33, 34]. The orthogeriatric co-management care has greatly improved the level of comorbidity management, which can stabilize and improve the general condition of long-lived patients better and faster, avoid ICU admission, and then reduce the early mortality of long-lived elderly hip fracture patients. Second, it was reported previously that the comorbidities of elderly hip fracture patients can be better evaluated and treated in the orthogeriatric co-management care, so as to achieve early surgery, which is likely to reduce the early mortality [18, 19, 28, 29]. And the proportion of surgery performed within 48 h of CM group was increased indeed in this study. In terms of the relationship between early surgery and outcome, this is still at the center of debate [12, 20, 21, 25, 27, 35–37]. Preoperative waiting time in the elderly patients with comorbidities might influence the outcome, as early surgery might prevent adverse events, such as pressure sores and pneumonia, but delayed surgery to optimize patients with comorbidities might improve their outcome [10, 25, 37]. Our experience suggests elderly patients with hip fractures should be performed an operation as soon as their medical condition permits. Third, better comorbidity management will undoubtedly increase the capacity of long-lived patients to tolerate surgery and anesthesia, which increases the chances of surgery, otherwise the prognosis of non-surgical treatment of long-lived elderly patients is really poor due to the high rates of mortality and morbidity [38, 39]. Non-surgical treatment only applies to the critically ill patients who cannot tolerate surgery or the patients has significantly reduced life expectancy [27, 40]. The proportion of patients undergoing surgery of CM group was higher in this study, which supports the discussion. Fourth, when orthopedic surgeons are detained in the operating room, if there is no orthogeriatric co-management care, the resolution of acute medical problems in the ward may be delayed. Instead, there are geriatricians to deal with patients’ acute medical problems. Last but not least, multidisciplinary geriatric teams with evidence-based experience are likely to provide considerable postoperative survival advantages for elderly hip fracture patients through specific geriatric interventions and early mobilization support, and they are more specialized in the secondary prevention of fractures (i.e., bone protection and falls assessment).

There are also differences in in-hospital mortality between the two groups, but there is no difference in 1-year mortality. This trend of mortality suggests that the co-management care made a difference in the early stage after fracture and has little impact on long-term mortality. This is in line with the study of Rapp et al. (2020) who reported the lower daily mortality rates in hospitals with orthogeriatric co-management were limited to the first 6 weeks after hospital admission [19]. The previous studies showed higher in-hospital mortality rates (6–11.6%%) [22–24, 41], most probably as a result of a shorter hospital LOS, and a better and faster comorbidity management in the orthogeriatric co-management care. There are also many studies showed a decrease in 1-year mortality [28, 30, 42]. This is not completely in concordance with the findings of this study, which may be explained by the fact that the target population of this study was the long-lived elderly patients ≥ 90 years old, who were generally in a serious condition and had a limited life expectancy. In a large database study of 11184 nonagenarian hip fracture patients undergoing surgery in Taiwan during the period 1997 and 2010, the 1-year mortality rate was 29.5% [43], which was higher than in this study (18.83). The difference is likely to be explained by the progress of national health care. The present study only observed patients in 2019, when the health care is undoubtedly more advanced than 1997–2010.

Previous studies used Barthel index or walking ability to assess the functional outcome of long-lived patients, 65–90% of patients can walk independently or with assistance after surgery, which is in concordance with this studies [41, 44–46]. And there were few studies to explore the effect of co-management care on the functional outcome of long-lived hip fracture patients. This study showed that co-management care can significantly improve the early functional outcome of these patients, which probably resulted from the combination of more meticulous comorbidity management and more comprehensive rehabilitation guidance after surgery can promote earlier functional recovery of patients. Besides, EQ-5D Questionnaire was used to assess patients’ HRQoL in this study. The CM group had a higher quality of life in the early stage after surgery, which tended to converge at 1 year, which is in line with the trend of functional outcome and also supports previous discussions. But there is no significant difference in 30-day EQ-VAS between the two groups, with an average of 66.78. This might be a result of the low self-demand of long-lived elderly patients for daily activities.

The hospital LOS was also significantly shortened under the co-management care, which was in line with the meta-analysis results of Annelore et al. [46]. The orthogeriatric co-management care advocates early discharge after patients’ condition is stable, so as to reduce patients’ medical expenses and improve the utilization rate of medical resources. The incidence of clinical adverse events is an important variable to evaluate the management level of hip fracture in the elderly. In previous studies, early surgery was often associated with a lower risk of clinical adverse events, like pressure sores, pneumonia, DVT and urinary tract infection, among elderly hip fracture patients [36]. There was no significant difference in the incidence of several clinical adverse events in this study. The reason for the results might be the general improvement of nursing level in China, especially in the Beijing region. The reported incidence rate of adverse events in literature ranged widely from 14.6 to 100%, which were obviously higher than in this study, most probably because of differences in the completeness of registration and definition of an adverse event [38, 47]. The 1-year reoperation rates were both low in the two groups, 1.6% and 1.0% respectively. This might be a result of the lower requirements for daily activities in long-lived elderly patients, and the lack of willingness of their families to reoperation. The previous studies from Taiwan in 1997–2010 showed a higher 1-year reoperation rate (7.31%) than the present study (1.35%) [43], which might be explained by the progress in internal fixation technology in the past 20 years. There was no significant difference in the total cost in two groups, which means that the orthogeriatric co-management care does not impose an additional economic burden on patients during this treatment.

This study is the first multicenter, prospective and controlled study to specially evaluate the efficacy of the orthogeriatric co-management care for the long-lived elderly hip fracture patients. It will provide important evidence for the promotion and the construction of orthogeriatric co-management care in China, and provide a direction for the future research on the orthogeriatric co-management care of elderly hip fracture in China. However, although this study used multivariate analysis to eliminate the confounding bias as much as possible, there are still some shortcomings: (1) without randomization, the differences in baseline data cannot be completely eliminated; (2) The CM group included only 1 hospital, while the TC group included 5 hospitals of different levels. There were biases caused by different levels of health service in the study; (3) The follow-up time is too short to provide long-term clinical efficacy; (4) The sample size is small. The results of this study must be validated in larger multicenter controlled studies with a longer follow-up period. Furthermore, this study only demonstrated that the orthogeriatric co-management care is better than traditional consultation mode in reducing mortality and improving functional outcomes, the optimal management mode still needs to be further explored.

## Conclusions

For long-lived elderly hip fracture patients, orthogeriatric co-management care lowered early mortality, improved early mobility and quality of life compared with the traditional consultation mode.

## Data Availability

The datasets used in this study are not publicly available because of patient confidentiality but are available from the corresponding author on reasonable request.
